# P-207. Implementation of Two-Step *C. difficile* Testing to Reduce HO-CDI and Antibiotic Use with Analysis of Ordering and Treatment Practices

**DOI:** 10.1093/ofid/ofae631.411

**Published:** 2025-01-29

**Authors:** Emma C Phillips, Brandi Manning, Sydney Agnello, Erica E Reed, Kelci E Coe, Nora Colburn, Shandra R Day

**Affiliations:** Ohio State University Wexner Medical Center, Columbus, Ohio; Ohio State University Wexner Medical Center, Columbus, Ohio; The Ohio State University, Columbus, Ohio; The Ohio State University Wexner Medical Center, Columbus, Ohio; The Ohio State University Wexner Medical Center, Columbus, Ohio; The Ohio State University Wexner Medical Center, Columbus, Ohio; Ohio State University Wexner Medical Center, Columbus, Ohio

## Abstract

**Background:**

*C. difficile* infection (CDI) is an ongoing challenge for healthcare facilities. The mischaracterization of *C. difficile* colonization as active infection falsely elevates healthcare onset-CDI (HO-CDI) rates and increases antimicrobial overuse and healthcare costs. Two-step testing for CDI helps differentiate colonization from infection. We describe the change in HO-CDI rates and antimicrobial use following implementation of two-step *C. difficile* testing with analysis of order and treatment practices post-implementation.

**Figure 1:**
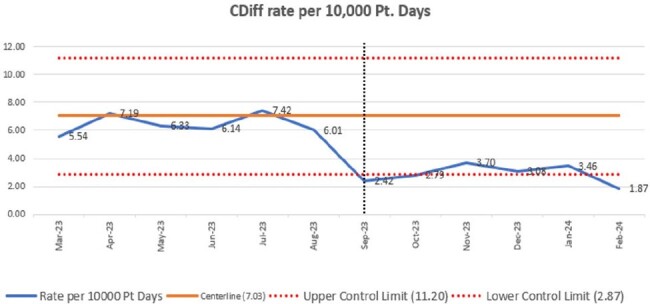
HO-CDI Rates Pre and Post-Intervention HO-CDI rates per 10,000 patient days pre and post-intervention. The vertical dotted black line indicates the date of intervention.

**Methods:**

Two-step *C. difficile* testing (PCR followed by ELISA for toxin if PCR-positive) was implemented Sept. 5, 2023. HO-CDI rates were compared for 6 months pre and post intervention using MedCalc statistical software. Antimicrobial patient days per 1000 patient days for oral vancomycin and fidaxomicin was compared pre and post-intervention using Wilcoxen Rank Sum test. Analysis of all inpatients with a *C. difficile* order during the post-intervention period was performed including test result, demographics, diarrhea documentation, laxative use, *C. difficile* treatment and repeat testing.

**Figure 2:**
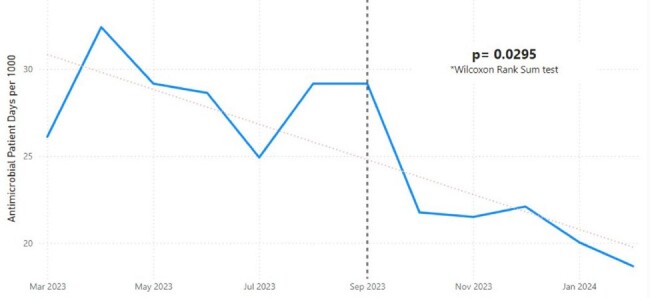
Antimicrobial Patient Days for PO Vancomycin and Fidaxomicin Pre and Post-Intervention Antimicrobial patient days per 1000 pre and post-intervention. The vertical dotted black line indicates the date of intervention.

**Results:**

HO-CDI rates decreased from 6.44 to 2.89/10,000 patient days pre and post-intervention with an incidence rate ratio of 2.22, P< 0.0001 (Figure 1). Inpatient prescriptions for oral vancomycin and fidaxomicin decreased from 28.4 to 22.2 antimicrobial patient days per 1000 patient days, p=0.0295 (Figure 2). A total of 1836 tests were done in the post-intervention period with detailed analysis included in Table 1. Less than 20% of patients had ≥3 diarrhea stools documented and excluding orders on HD 1 or 2, stool documentation was present in 29% of patients. Analysis of the 66 HO-CDI cases was similar compared to all positive cases with 24 (36%) receiving laxatives and 21 (36%) with diarrhea documented.

**Table 1:**
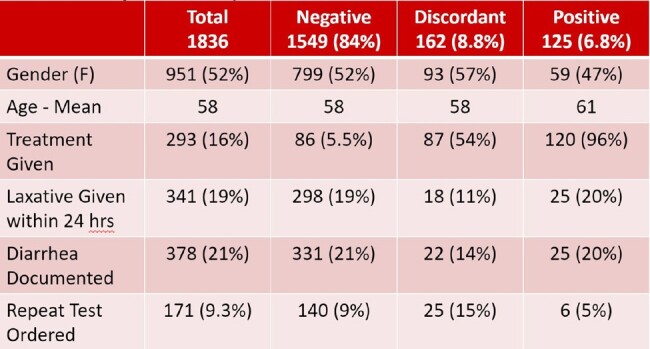
Analysis of All Inpatient C. diff Orders Post-Intervention Analysis of all inpatient C. diff orders post-intervention, where negative indicates PCR negative / toxin negative; discordant indicates PCR positive / toxin negative; and positive indicates PCR positive / toxin positive.

**Conclusion:**

HO-CDI rates and *C. difficile* specific antimicrobial use were significantly decreased after implementation of two-step testing. Despite this decrease, over half of discordant result patients received *C difficile* treatment, indicating an opportunity for additional education. Optimization of diagnostic stewardship with regards to laxative use and appropriate stool documentation are areas of opportunity.

**Disclosures:**

**All Authors**: No reported disclosures

